# The Predictors of Unsafe Behaviors among Nuclear Power Plant Workers: An Investigation Integrating Personality, Cognitive and Attitudinal Factors

**DOI:** 10.3390/ijerph20010820

**Published:** 2023-01-01

**Authors:** Da Tao, Xiaofeng Diao, Xingda Qu, Xiaoting Ma, Tingru Zhang

**Affiliations:** Institute of Human Factors and Ergonomics, College of Mechatronics and Control Engineering, Shenzhen University, Shenzhen 518060, China

**Keywords:** unsafe behaviors, personality traits, executive function, safety attitudes, nuclear power plants

## Abstract

Unsafe behaviors, such as violations and human errors, have long been recognized as the main causes of accidents in nuclear power plants (NPPs). However, personal factors that influence unsafe behaviors among NPP workers have not been well examined, especially in an integrated model. This study proposes an integrated contextual mediated model to examine personality, cognitive and attitudinal predictors of unsafe behaviors among commissioning workers at NPPs. The model was verified using structural equation modeling technique with survey data from 177 commissioning workers in two Chinese NPPs. Results show that personality traits (i.e., conscientiousness, neuroticism, openness and agreeableness), executive function and safety attitudes exerted significant effects on unsafe behaviors. In addition, the effects of agreeableness and executive function were partly mediated by safety attitudes. Both conscientiousness and neuroticism indirectly influenced unsafe behaviors through the mediating role of executive function. The findings shed light on the design of evidence-based interventions for safety performance in NPPs.

## 1. Introduction

Nuclear safety represents a significant concern worldwide [1]. Violation of nuclear safety can lead to severe consequences, such as nuclear accidents and disasters, as well as tremendous damage to the economy and society. For example, as two of the largest nuclear accidents in the history, the Three Mile Island accident in 1979 cost one billion US dollars for clean-up and led to the stagnation of the United States nuclear industry for 30 years [2], while the Chernobyl nuclear accident in 1986 led to 6000 childhood cases of thyroid cancer immediately after the accident and around 25,000 cumulative cancer fatalities in the affected areas until 2011 [3].

Given the severely negative consequences of nuclear accidents, the investigation of their causes and prevention strategies has attracted much research attention [4,5,6]. One of the main causes of accidents in NPPs is unsafe behaviors, which may arise from one’s intended or unintended actions in contravention of safety regulations and procedures (i.e., human errors and violations). In fact, unsafe behaviors have always been recognized as one of leading factors that threaten nuclear safety [7,8]. According to event analysis reports by the World Association of Nuclear Operators (WANO), unsafe behavior-induced human errors were associated with 59% of the 940 nuclear accidents that occurred between 1993 and 2002 worldwide [9]. The statistic is similar in China, where 65% of nuclear accidents reported from 2003 to 2010 were related to human errors [4].

Evidence has shown that a large number of human errors and accidents are experienced by certain groups of workers [10]. This suggests that there are personal-related factors that predispose certain workers, rather than other workers, to perform unsafe behaviors and be involved in accidents. Previous studies have proposed that personality traits, cognitive abilities (e.g., executive function) and attitudinal factors (e.g., safety attitudes) may be important personal factors related to unsafe behaviors in varied occupational and non-occupational contexts [11,12,13,14,15,16]. However, research gaps must be addressed before one can apply the research evidence to NPP contexts. First, substantial variability exists in the influence of these personal factors in different occupational and non-occupational settings [11,17,18]. This suggests that their influence could be context-specific, and the contexts in which the accidents and unsafe behaviors occur need to be well considered. However, most of prior evidence has come from such domains as driving, aviation, mining and construction industries [12,19,20,21,22]. Such evidence might not be applicable at NPPs due to the domain difference. This is because NPPs are uniquely characterized by safety-critical features, complex work procedures, high work pressure, and tremendous damage to the economy and society resulting from nuclear disasters [5,23]. Thus, workers at NPPs may perceive issues and behave differently from people in other domains. Second, work at NPPs involves a number of cognitive activities concerning a great deal of information and complex procedures. While cognitive abilities (e.g., executive function) can be especially important in the workplace [13], their role in unsafe behaviors have been less examined. Finally, evidence shows that personality traits, executive function and safety attitudes closely correlate with each other [24,25,26], but few efforts have been made to explore an integrated model of unsafe behaviors with these factors, especially in NPP contexts. How these factors interact with each other to affect unsafe behaviors appears unknown.

China has placed nuclear power technology at the national strategy level, and is recognized as one of the leading countries to develop NPPs [27]. The development of NPPs in China has inspired research efforts on associations between personal factors and unsafe behaviors, especially in the commissioning phase, as commissioning is a safety-critical phase in the NPP lifecycle that aims to diagnose and fix possible deficiencies and errors before the commercial operation of NPPs [28,29]. In particular, commissioning workers usually encounter large challenges in their work compared with workers in main control rooms [29], such as high mental workload, unexpected emergencies, multi-tasking requirements, and difficult work environments. Thus, they are more susceptible to unsafe behaviors, which are likely to cause a range of consequences, such as device malfunction, excessive radiation leakage, and even nuclear accidents [30]. Thus, this study aimed to examine personality, cognitive and attitudinal predictors of unsafe behaviors among commissioning workers at NPPs through an integrated contextual mediated model.

## 2. Literature Review 

### 2.1. Unsafe Behaviors

Safety outcomes in safety research are usually indicated by the frequency of accidents and injuries and safe/unsafe behaviors [31]. Accidents and injuries usually occur at low frequencies and require extensive cost and time for data collection, making them less preferred indicators of safety outcomes. Previous researchers have mostly used safe/unsafe behaviors as safety outcomes due to the ease of measuring them. While safe behaviors concern safety outcomes from a positive perspective, unsafe behaviors are more relevant to the occurrence of accidents and injuries, and thus were examined in this study. Unsafe behaviors can be described as the contravention of safety practices, such as violations of safety regulations and failures to follow planned actions [8]. A previous review reported that 76% of industrial accidents in the USA were caused by workers’ unsafe behaviors [32]. Two typical unsafe behaviors are violations and human errors, the difference between which depends on whether they are caused by intended actions. [8,33]. Violations refer to intentional risky behaviors that may present threats to safety [33], while human errors refer to unintended behaviors that fail to complete planned actions due to misjudgment or failure of observation, and could create potential unsafe outcomes [33].

### 2.2. Personality and Unsafe Behaviors

Personality traits can be described as stable and consistent behavioral patterns that a person has, and remain stable in different times and situations [34]. Researchers in the 1960s studied the role of personality traits in unsafe behaviors among drivers and minors [35,36]. It was found that extroverted motor vehicle drivers were involved in more traffic violations and accidents than introverted drivers [35]. Expediency was significantly related to the number of violations among juvenile drivers [36]. Later studies were conducted to explore relationships between personality traits and unsafe behaviors in various industries [37,38]. For example, Hansen, in a review study, concluded that the occurrence of many industrial and traffic errors and accidents was associated with such personality traits as extroversion, locus of control, impulsivity, aggression, and neurosis [37]. Lardent [38] identified five personality factors (i.e., conscientious, suspicious, shrewd, self-sufficient and tense) that underlie accident-prone behaviors in fighter pilots. Other studies found that individuals with negative personality traits, such as emotional instability, impulsiveness, risk-taking, depression and anger, were more likely to show risky behaviors and be engaged in accidents [39,40,41]. Neuroticism and psychoticism were also shown to be predictors of risky driving behaviors [19]. 

The Big Five Personality Model [34] is a well-known assessment tool for personality traits and was adopted in our study. It divides personality into five dimensions, including extroversion, agreeableness, conscientiousness, neuroticism, and openness to experience. Its reliability and validity have been widely verified in a number of occupational settings [17,42,43]. Numerous studies have examined Big Five personality dimensions and their relationships with unsafe behaviors and accidents [17]. Several review studies have reported that low conscientiousness and low agreeableness are valid in the prediction of accident involvement, especially for occupational accidents, while extroversion and openness appeared to exert little effects on occupational accidents [12,17]. In contrast, other reviews reported varied effects on unsafe behaviors and workplace accidents for some personality traits, except that low agreeableness was shown to be a valid predictor for workplace accidents [11]. All the reviews have pointed out that accident contexts may act as moderators in personality-accident relationships [11,12,17].

### 2.3. Executive Function and Unsafe Behaviors

Executive function is among important cognitive abilities related to safety behaviors and accidents [13]. Executive function refers to a set of basic and complex cognitive processes (e.g., decision-making, multitasking, behavioral disinhibition, strategic planning, organization and problem solving), through which individuals can adjust their thoughts and actions in a variety of circumstances [24,44]. Thus, executive function is vital for behavioral performance and successful adjustment to unexpected circumstances in complex work conditions [13]. 

While many studies have examined associations between executive function and safe/unsafe behaviors, their results seem mixed [18,44,45]. For example, León-Domínguez et al. [46] found that executive function is positively associated with safe driving behaviors among middle-aged drivers. Low executive function is more likely to result in dangerous driving behaviors and negative driving outcomes among college students [47]. Mäntylä et al. [48] observed that poorer executive function led to more errors in simulated driving tasks. Pharo et al. [49] reported that executive function was associated negatively with various real-life risk-taking behaviors (e.g., abuse of alcohol and drugs, antisocial and unsafe sexual behavior, and risky driving). In contrast, some studies indicated that better executive function resulted in increased risky driving behaviors among young and older drivers [50,51], as drivers high in executive function may overestimate their abilities to deal with the unforeseen consequences of their actions. Other studies even found no association between executive function (e.g., inhibitory control) and risky driving behaviors [48]. Moreover, executive function was also shown to exert its effects on risky behaviors through attitudes towards risky behaviors [14].

While executive function can be especially important for occupational functioning and work performance in the workplace, few studies have made endeavors to investigate its role in unsafe behaviors among occupational settings. Moreover, previous studies focused more on basic cognitive processes (e.g., working memory, attention, and inhibitory control). In contrast, it is believed that some higher levels of executive function (e.g., strategic planning and organization) can be more important in complex occupational settings such asNPPs, as work in these settings requires the capacity for cross-temporal organization, problem-solving, and planning toward goals for a longer period, such as days, weeks, and months [13]. Finally, recent studies have shown that executive function correlated with personality and may mediate the role of personality traits in unsafe behaviors [14,49]. Therefore, the role of higher levels of executive function in unsafe behaviors deserves exploration.

### 2.4. Safety Attitudes and Unsafe Behaviors

Safety attitudes refer to one’s attitudinal beliefs and commitments regarding safety procedures, policies and practices within organizations [52]. Numerous studies have suggested that safety attitudes could influence safe or unsafe behaviors [21]. For example, Iversen investigated three types of safety attitudes among drivers, and found that attitudes towards drinking and driving, attitudes towards others’ careless driving, and attitudes towards rule violations were related to safe driving behaviors [53]. Moreover, safety attitudes were shown to mediate the relationships between personality traits, executive function and safe behaviors [25,43,54]. For instance, Ulleberg and colleagues found that attitudes towards traffic safety exerted a significant mediating role in the relationships between personality traits and risky behaviors during driving [54]. The mediating role of safety attitudes has also been verified by more recent studies on elevator workers [25].

### 2.5. Research Model

**Figure 1:**
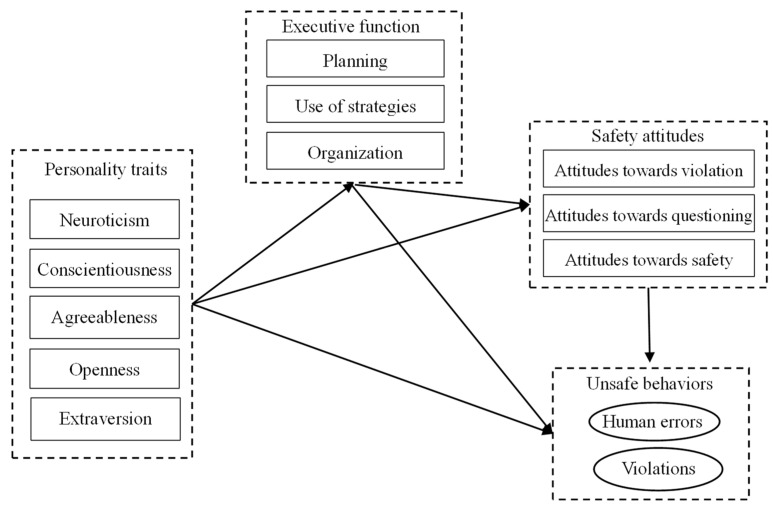
The contextual mediated model proposed in this study.

A contextual mediated model (Figure 1) was therefore proposed based on the literature review presented above to examine the roles of personality traits, executive function and safety attitudes in unsafe behaviors in NPP contexts. It was hypothesized that personality traits and executive function would influence unsafe behaviors, and the influence could be mediated by safety attitudes. In addition, personality traits would also exert indirect effects on safety attitudes and unsafe behaviors through the mediating role of executive function. 

## 3. Methods

### 3.1. Study Samples and Procedures

This study adopted a questionnaire survey method for data collection, which is a widely used and legitimate approach to explore individuals’ psychological and behavioral responses [55]. Study samples were recruited from two NPPs in South China, which were affiliated with China General Nuclear Power Corporation (CGN). CGN is the largest nuclear power operator in China and is also one of the largest nuclear power constructors worldwide [56]. Two NPPs were chosen as they were new NPPs in construction (otherwise, there was no commissioning work) and had similar organizational culture, work regulations and environmental conditions.

Commissioning workers from the two NPPs were invited to complete a paper-based questionnaire. A key contact person at the NPPs served as the link between researchers and participants, and helped the researchers send questionnaires to the workers. All commissioning workers at the two NPPs were encouraged to participate in the survey to obtain sufficient and representative samples, and to reduce possible sampling error. After the workers provided informed consent, they were asked to complete the questionnaires on their own, and could ask the researchers for explanation if necessary. In order to minimize potential biased responses, at the time of recruitment, participants were explicitly informed that they were allowed to anonymously complete the questionnaires and should answer the questionnaires carefully and honestly; that their information would be confidentially kept and be de-identified before it was used for research purpose, and that they were required to directly return the questionnaires to the researchers’ offices that were temporarily located at the NPPs. One hundred and seventy-nine questionnaires were returned (response rate: 82%), among which, 177 were valid samples and kept for data analysis (two incomplete samples were deleted).

### 3.2. Instruments

The questionnaire was designed based on a thorough literature review and was refined by a cognitive interview with 12 experienced NPP staffs. The cognitive interview was conducted to improve the questionnaire’s clarity and reliability [57]. Three safety research experts were also consulted to improve the quality of questionnaire. As a commonly used practice [58], both positively and reversely worded items were included in the questionnaire to reduce possible response bias. The questionnaire consisted of items to collect participants’ demographic information (e.g., age, gender, education, and work experience), and their responses on personality traits, executive function, safety attitudes and unsafe behaviors. The items for the constructs examined in the proposed model (Table 1) were answered on 5-point Likert scales, with agree-disagree anchors for personality traits, executive function and safety attitudes, and with never-always anchors for unsafe behaviors. Average scores for each of the constructs were used in analyses.

#### 3.2.1. Personality Traits

Personality traits were examined with the Big Five personality model [34], and measured by a valid Chinese version of Big Five Personality Inventory (CBF-PI), which has been demonstrated to have adequate reliability among Chinese population [59]. The CBF-PI consists of five 5-item scales that assess the five personality traits accordingly.

#### 3.2.2. Executive Function

Executive function was assessed by the Executive Function Index developed by Spinella [60] (Table 1). Based on results of our exploratory factor analysis, a three-factor solution was obtained from the Index, which could explain 55.2% of the variance. The three factors were named as planning (four items), use of strategies (three items) and organization (five items). Planning referred to individuals’ tendency to plan and think ahead to deal with work. Use of strategies reflected individuals’ abilities to use strategies in learning and recovering from mistakes. Organization measured individuals’ ability to perform organized and goal-directed behaviors [60].

#### 3.2.3. Safety Attitudes and Unsafe Behaviors

Safety attitudes were assessed by an 11-item Safety Attitudes Questionnaire developed based on risk-taking attitudes scale from previous studies [53] and scales currently used in practice of the examined NPPs (Table 1). Based on results of our exploratory factor analysis, a three-factor solution was obtained from the questionnaire, which could explain 68.6% of the variance. The three factors were named as attitudes towards violation (four items), attitudes towards questioning (three items) and attitudes towards safety (four items). Higher scores indicated more positive attitudes towards violation (i.e., favor of no violation), questioning and safety. The average scores for each of the three attitudes were used in analyses.

Two types of unsafe behaviors were examined: human errors and violations. Human errors were measured with a seven-item scale adopted from previous studies [7,61]. A three-item scale from a previous study [7] was used to assess violations. The two scales demonstrated adequate reliability among nuclear power plant operators [7].

### 3.3. Data Analysis

Data cleaning was first performed by excluding respondents who provided incomplete, unrealistic or inconsistent responses. The inter-correlations among major variables were examined with Pearson’s correlation analysis. Reliability, convergent validity and discriminant validity of the measurements were examined to verify the measurement model. In particular, reliability was assessed by Cronbach’s alpha, and is considered as good and acceptable if Cronbach’s alpha is greater than 0.7 and 0.65, separately [62]. Convergent validity is achieved if factor loadings for construct items are greater than 0.5, the average variance extracted (AVE) for each construct is larger than 0.5 and composite reliability for each construct exceeds 0.7 [63,64]. Discriminant validity is confirmed if the square root of AVE for each construct is larger than all of its correlations with any other constructs [63,64]. In addition, the severity of multicollinearity among variables was assessed by tolerance and variance inflation factor (VIF) values. Multicollinearity was considered present if tolerance values were less than 0.10 and VIF values were larger than 5.0 for the variables. The structural model was examined by structural equation modeling (SEM) analysis methods with AMOS 21. The overall model fit was assessed by commonly used goodness-of-fit indices with criteria set as the ratio of χ2 to degrees of freedom (χ2/df < 3), the incremental fit index (IFI > 0.90), the Tucker Lewis index (TLI > 0.90), the comparative fit index (CFI > 0.90), and root mean square error of approximation (RMSEA < 0.08) [65,66]. Multiplicity control was applied using Benjamini and Hochberg’s false discovery rate controlling step-up Bonferroni (FDR) procedures to control the rate of type I errors, which could be possibly induced by multiple hypothesis tests in the SEM [67,68].

## 4. Results

### 4.1. Descriptive Results

Most samples were males (98.3%), and three were female (1.7%). They had an average age of 32.6 years (SD = 5.1), with 83.5% of participants aged between 28 and 36. The respondents were well educated, with bachelor’s degree (85.9%), college degrees (5.6%) and master’s degree (8.5%). Most of them were married (72.9%), while the rest were single (27.1%). The mean (SD) working experience at NPPs was 7.7 (2.4) years, and 96% of the respondents had working experience ranging from 5 to 13 years.

### 4.2. Model testing

#### 4.2.1. Measurement Model

The Cronbach’s alpha of measurement scales ranged from 0.68 to 0.87 (Table 1), indicating acceptable reliability of the measurements. Table 2 shows the convergent validity results. Factor loadings for the construct items ranged from 0.60 to 0.87, greater than 0.5. The composite reliability ranged from 0.80 to 0.90, greater than 0.70. The AVEs for all constructs were larger than 0.50. Thus, convergent validity was achieved. Table 3 shows that the square root of the AVE for each construct was greater than any of the correlations involving the construct. Therefore, discriminate validity was confirmed. Table 4 shows that VIF values ranged from 1.204 to 1.712, and tolerance values ranged from 0.584 to 0.831. Therefore, multicollinearity was considered absent for the examined variables.

#### 4.2.2. Structural Model

Initial SEM analysis was conducted to estimate the proposed structural model. However, feedback from the modification indices indicated that several Big Five personality dimensions were highly correlated with each other (e.g., Conscientiousness-Agreeableness, and Openness-Extroversion) and should be considered in the model. Thus, we added these correlations into the model and re-analyzed the updated model with SEM. The indices showed a good fit between the examined model and the data (χ^2^/df = 1.487, RMSEA = 0.053, CFI = 0.924, IFI = 0.927, TLI = 0.910).

**Figure 2:**
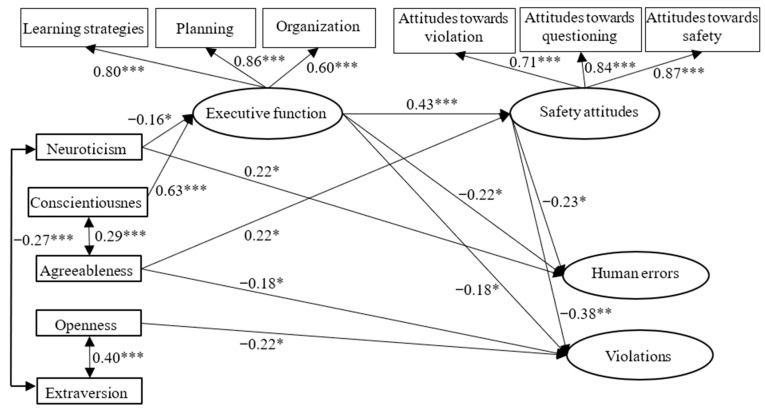
Results for the final model with significant structural relationships. * *p* < 0.05; ** *p* < 0.01; *** *p* < 0.001.

Figure 2 shows the results for significant structural relationships in the estimated model. In the model, conscientiousness positively predicted executive function (*β* = 0.63, *p* < 0.001). Agreeableness positively affected safety attitudes (*β* = 0.22, *p* = 0.018) and negatively correlated with violations (*β* = −0.18, *p* = 0.045). Openness was negatively related to violations (*β* = −0.22, *p* = 0.017). Neuroticism negatively correlated with executive function (*β* = −0.16, *p* = 0.044) and exerted a direct impact on human errors (*β* = 0.22, *p* = 0.013). Executive function positively predicted safety attitudes (*β* = 0.43, *p* < 0.001), while it negatively correlated with human errors (*β* = −0.22, *p* = 0.039) and violations (*β* = −0.18, *p* = 0.045). In addition, safety attitudes negatively affected human errors (*β* = −0.23, *p* = 0.048) and violations (*β* = −0.38, *p* = 0.007). Extroversion showed no direct effects on executive function, safety attitudes and unsafe behaviors. Conscientiousness explained 42% of variance in executive function. The amount of variance in safety attitudes accounted for by executive function and agreeableness was 27%. Safety attitudes and executive function explained 22% of the total variance in human errors. Agreeableness, openness, executive function and safety attitudes explained 40% of the total variance in violations.

## 5. Discussion

This study is one of the first studies examining personality, cognitive and attitudinal predictors of unsafe behaviors among NPP workers with an integrated contextual mediated model. The results demonstrate the predictive role of personality (i.e., neuroticism, conscientiousness, agreeableness and openness), executive function and safety attitudes in unsafe behaviors.

### 5.1. Primary Findings

One of important contributions from this study is that it showed and clarified the predictive role of executive function and several personality traits in safety attitudes and unsafe behaviors at NPPs, while at the same time it demonstrated the mediating role of safety attitudes in the links among personality traits, executive function and unsafe behaviors. In addition, compared with what was reported in previous studies [19,25,69], we found that a higher percentage of variance in unsafe behaviors (40% for violations) could be explained by executive function, personality traits, and safety attitudes. One explanation would be that, different from previous studies, our study additionally integrated an important cognitive ability (i.e., executive function) in the model, which was verified to be an important predictor of unsafe behaviors. This may lead to a higher percentage of explained variance. In contrast, many of previous studies investigated a variety of personality traits in relation to unsafe behaviors [19,25,54], but few examined personality-unsafe behaviors associations in combination with cognitive abilities [14,26]. While our study confirmed some well-documented personality traits-unsafe behaviors associations, it also provided new evidence on how executive function interacted with personality traits and safety attitudes to affect unsafe behaviors in NPP contexts.

Previous studies mostly examined basic executive functions (e.g., attention, memory and impulse control) that are more important in quick response and short-term behavioral control (e.g., driving behaviors) [14,18,45,46,48,51]. In contrast, this study examined higher levels of executive function (i.e., planning, organization and use of strategies) that could be essential to successful completion of complex work at NPPs, as these executive function measures are believed to meet requirements on both short-term and long-term organization, planning and problem-solving toward effective and safety performance in complex occupational settings [13]. We found that executive function could predict safety attitudes and unsafe behaviors. In particular, executive function had direct negative effects on both human errors and violations, while it also affected human errors and violations through the mediating role of safety attitudes. It may be that workers higher in executive function are good at planning, organizing and executing tasks with complex procedures, and thus are more likely to prevent their behaviors from errors and violations. It is also intuitive that people good at strategic planning would plan, think ahead and use strategies to deal with work. Thus, they show more tendency to develop positive and serious attitudes towards safety before they perform daily tasks. Therefore, executive function can be an important cognitive ability that is able to prevent workers from human errors and violations.

Our finding showed that conscientiousness was positively related to executive function. This is in line with previous studies [24,26]. This implies that conscientiousness affects safety attitudes and unsafe behaviors indirectly through executive function. It is reasonable that conscientious individuals are more inclined to plan ahead and do things more logically and in an organized manner. The results indicate that a conscientious personality can be important at NPPs, as work there requires strict compliance with regulations and rules, violation of which can lead to great threats to workplace safety.

The results showed that agreeableness affected unsafe behaviors both directly and indirectly through the mediating role of safety attitudes. This is similar to previous studies [11,14,17], which suggested that agreeableness is a generalizable predictor of safety behaviors and accidents in both occupational and non-occupational scenarios. It could be that commissioning work at NPPs involves many team-based tasks, where organizational regulations and rules serve as group norms for safe behaviors, and would have significant impacts on error involvement. People with high scores in agreeableness tend to adhere to group norms. Previous studies also found that agreeableness had the most influence on job performance involving team-based working [70]. People with low agreeableness were more likely to perform interpersonal violations [71]. This appears to indicate that teams with more agreeable individuals would be more likely to comply with safe behaviors, thereby leading to fewer errors.

Openness has been a less examined personality dimension in previous studies in relation to safe/unsafe behaviors and accident involvement compared with other personality traits. Our study found that openness was negatively related to violations. This finding is inconsistent with previous studies [11,17]. It seems counterintuitive and supports an unusual expectation that curious, imaginative and unconventional individuals are more liable to compliance of rules and regulations. The finding might be mediated by some uninvestigated factors, such as work environment. For example, commissioning work at NPPs involves a number of team-based tasks, such as communication, negotiation and coordination, and thus individuals open to experience may perform better in such tasks.

There was no effect of extroversion on either safety attitudes or unsafe behaviors. Previous evidence on the relationships between extroversion and safe behaviors has been mixed. They could be negative [35,37], non-existent [14,19,72], or positive [42]. In fact, several previous reviews have concluded that extroversion is more likely to be a valid predictor for risky behaviors and accidents in non-occupational contexts (e.g., driving), instead of occupational contexts [11,12,17]. It is speculated that the association between extroversion and unsafe behaviors might be moderated by the type of task undertaken. Previous studies from driving psychology have shown that extroverts would be more likely to take risky behaviors when performing tasks that are monotonous or require sustained attention [17]. In contrast, commissioning work is more complex and challengeable, and thus is less likely to be affected by extroversion [72]. More efforts are needed before firm conclusions can be drawn on the role of extroversion in complex task contexts.

Importantly, neuroticism exerted a positive effect on human errors directly and indirectly through the mediating role of executive function. Consistently, previous studies have predominately reported that neuroticism was related to risky behaviors, and could lead to more errors [12,17,19]. The reason why neuroticism presents as a risky personality trait may be that neurotic people are usually preoccupied by worry and anxiety, and can have difficulties in concentrating on their tasks, thereby resulting in a high likelihood of human errors [19]. Therefore, neuroticism is a significant concern in safety management and should be paid close attention to in employee recruitment at NPPs.

### 5.2. Implications

Our study has important implications. Theoretically, our findings demonstrate the generalizability of the mediated “personality-safety attitudes-safety behaviors” model in NPP commissioning domain, which has also been verified in other domains [19,25,43]. In addition, our study integrated executive function in the mediated model, and verified its important role in shaping safety attitudes and unsafe behaviors. While the integration of such executive function measures into the mediated model appears encouraging, there are a number of other executive functions (e.g., response inhibition, interference control and mental set shifting) that might be associated with safe behaviors and human errors [18,73] and could be further examined.

In practice, findings from our study can benefit the selection of employees and help with the development of safety education and promotion interventions at NPPs. First, in line with long-term advocating to carry out psychological selection in the nuclear industry [74], NPP companies should assess the applicants’ personality and executive function in the recruitment process and guarantee that suitable individuals are employed so that possible unsafe behaviors can be minimized from human side. Individuals with high conscientiousness and agreeableness and higher levels of executive function should be given high priority in NPP worker recruitment, as they tend to show more serious attitudes towards safety, present less unsafe behaviors, and make fewer errors. In addition, the evidence obtained in our study could also aid the design of education and training programs for both experienced and novice commissioning workers by reinforcing their awareness of how their own personality traits and executive function could affect their daily work behaviors. Moreover, safety education interventions could also include cognitive training strategies to improve workers’ executive function [75]. Finally, safety promotion programs at NPPs should be aimed at reinforcing workers’ safety attitudes. Content of safety attitudes training and promotion could include zero tolerance to violation, higher priority of safety, and questioning of others’ unsafe behaviors and violations to regulations.

### 5.3. Limitations and Future Studies

This study has several limitations. First, our study samples were recruited from the commissioning division of NPPs. Whether the obtained evidence can be generalizable to other divisions (e.g., operation departments) awaits confirmation, as they have different work content and procedures. Future studies could include diverse samples from NPPs to address this limitation. Second, this study adopted self-reported data on unsafe behaviors, which may be susceptible to under-reporting because of participants’ concern about negative influence of their responses on their benefits. Although self-reported data have been widely used and shown to be correlated with objective measures [76], event reports and work performance logs at NPPs, if ethically and practically possible, can be extracted to provide objective data as complementary measures for unsafe behaviors.

## 6. Conclusions

The present study enhanced our understanding of a comprehensive set of predicators of unsafe behaviors among commissioning workers at NPPs. We examined the role of personality, executive function and safety attitudes by testing their associations with unsafe behaviors in a mediated model. The findings demonstrate that several personality traits (i.e., neuroticism, conscientiousness, agreeableness and openness) and executive function exerted significant effects on unsafe behaviors directly and indirectly through the mediating role of safety attitudes. In addition, conscientiousness and neuroticism interacted with executive function to affect safety attitudes and unsafe behaviors. The findings emphasize the important role of personality and executive function in the development of unsafe behaviors among commissioning workers, and provide implications in the development of evidence-based safety promotion and education interventions at NPPs.

## Figures and Tables

**Table 1:** Constructs items in the study.

Constructs	Scale Items	Sources	Cronbach’s Alpha
** Personality traits **			
Neuroticism	Neu1. I often worry that something bad will happen.	Wang et al., 2011 [59]	0.82
	Neu2. I often feel afraid.		
	Neu4. I come near to a breakdown in front of pressure.		
	Neu5. I often worry about something unimportant.		
	Neu6. I often feel upset.		
Conscientiousness	Con1. I always try my best in my work.	Wang et al., 2011 [59]	0.78
	Con2. I will persist in achieving my goal, once I have set it up.		
	Con3. I make decisions after careful consideration.		
	Con4. I am an organized and logical person.		
	Con5. I like to plan things out right from the beginning.		
Agreeableness	Agr1. I believe the essential goodness of human nature, even if there are some dark things in our society (such as wars, evil, and frauds).	Wang et al., 2011 [59]	0.68
	Agr2. I do not care much about whether others are treated unfairly. *		
	Agr3. I often think that the suffering of others does not concern me. *		
	Agr4. I often feel sorry for those who suffer misfortune.		
	Agr5. I am a person who just cares about myself and do not care about others. *		
Openness	Open1. I am an imaginative person.	Wang et al., 2011 [59]	0.82
	Open2. I am a curious person.		
	Open3. I like adventure.		
	Open4. I am a person who dares to take risks and break conventional rules.		
	Open5. I am eager to learn new things, even if these things have nothing to do with my daily life.		
Extraversion	Ext1. I enjoy attending social and recreational parties.	Wang et al., 2011 [59]	0.80
	Ext2. People often consider me passionate and friendly.		
	Ext3. I often act proactively and have fun in busy parties.		
	Ext4. There will be no silence if I am present.		
	Ext5. I like to be a leader rather than a follower.		
** Executive function **			
Planning	Plan1. Organized person.	Spinella, 2005 [60]	0.76
	Plan2. Save money regularly.		
	Plan3. Plan for the future.		
	Plan4. Anticipate consequences of actions.		
Learning strategies	LS1. Self-monitor for mistakes.	Spinella, 2005 [60]	0.70
	LS2. Use memory strategies.		
	LS3. Learn from mistakes.		
Organization	Org1. Trouble summing information for decisions. *	Spinella, 2005 [60]	0.70
	Org2. Distractibility. *		
	Org3. Lost track of what I am doing. *		
	Org4. Mix up the sequences of actions. *		
	Org5. Trouble doing two things at once. *		
** Safety attitudes **			
Attitudes towards violation	AV1. It is acceptable to ignore regulations or rules to proceed faster. *	Iversen, 2004 [53]	0.70
	AV2. Some operations can be omitted because of the strict rules. *		
	AV3. Operation rules are too complicated to be fully followed in practice. *		
	AV4. It is acceptable to disobey some operation rules when things are in good condition and no one else is around. *		
Attitudes towards questioning	AQ1. Co-workers should question each other only if there is something wrong, regardless of their positions.	Iversen, 2004 [53]	0.71
	AQ2. I should question co-workers’ behaviors if such behaviors are considered to present threat to safety.		
	AQ3. I should suspend the work when uncertainty exists.		
Attitudes towards safety	AS1. Safety has the highest priority when I make decisions in my work	Iversen, 2004 [53]	0.87
	AS2. It is my responsibility to comply with work procedures and regulations.		
	AS3. I pay particular attention to nuclear safety at work.		
	AS4. It is not allowed to take shortcuts when performing work.		
** Safety behaviors **			
Violations	Vio1. Sometimes I do not use all the necessary safety equipment to do my work.	Rao et al., 2017 [7]	0.73
	Vio2. Sometimes I do not follow correct safety procedures to do my work.		
	Vio3. Sometimes I do not ensure the highest levels of safety when I do my work.		
Human errors	HE1. Forgot to change the indicator of component status in the process chart.	Kecklund and Svenson, 1997 [61]; Rao et al., 2017 [7]	0.86
	HE2. Promised to return to someone with information but forgot to do so.		
	HE3. Forgot to perform an operation in a sequence of operations that I planned to carry through.		
	HE4. Was forced to interrupt task on hand because I was disturbed by someone and forgot to return to what I was busy doing.		
	HE5. I misunderstood the situation and made mistakes.		
	HE6. I missed important information because I didn’t concentrate.		
	HE7. When I was performing an operation, I was not able to remember all important information at the same time and had to ask for related information again.		

* Items reverse coded.

**Table 2:** Factor loading, AVE and composite reliability of the constructs.

Constructs	Items	Factor Loading	AVE	Composite Reliability
Executive function	Plan	0.86	0.58	0.80
	Org	0.60		
	LS	0.80		
Safety attitudes	AV	0.71	0.66	0.85
	AQ	0.84		
	AS	0.87		
Violations	Vio1	0.79	0.65	0.85
	Vio2	0.83		
	Vio3	0.79		
Human errors	HE1	0.77	0.55	0.90
	HE2	0.77		
	HE3	0.76		
	HE4	0.75		
	HE5	0.73		
	HE6	0.72		
	HE7	0.70		

Note: AVE: average variance extracted.

**Table 3:** Means of and correlations among major variables.

	1	2	3	4	5	6	7	8	9
1. Neuroticism	-								
2. Conscientiousness	−0.17 **	-							
3. Agreeableness	−0.29 **	0.33 **	-						
4. Openness	−0.11	0.34 **	0.17 *	-					
5. Extroversion	−0.32 **	0.25 **	0.27 **	0.45 **	-				
6. Executive function	−0.27 **	0.55 **	0.38 **	0.33 **	0.34 **	**0.762**			
7. Safety attitudes	−0.18 *	0.34 **	0.31 **	0.09	0.17 *	0.39 **	**0.812**		
8. Errors	0.30 **	−0.27 **	−0.19 *	−0.11	−0.16 *	−0.36 **	−0.34 **	**0.806**	
9. Violations	0.12	−0.32 **	−0.36 **	−0.30 **	−0.21 **	−0.39 **	−0.42 **	0.23 **	**0.742**
Mean	2.46	3.22	3.78	3.41	3.25	3.69	4.04	3.85	4.04
SD	0.67	0.42	0.48	0.59	0.55	0.36	0.46	0.49	0.59

The values on the diagonal (in bold) are the square root of AVE. * *p* < 0.05; ** *p* < 0.01.

**Table 4:** Multicollinearity analysis of variables.

Variables	Tolerance	Variance Inflation Factors
1. Neuroticism	0.831	1.204
2. Conscientiousness	0.643	1.556
3. Agreeableness	0.779	1.284
4. Openness	0.726	1.377
5. Extroversion	0.701	1.427
6. Executive function	0.584	1.712
7. Safety attitudes	0.791	1.265

## Data Availability

Data are available on request from the corresponding author.
